# Bile Acids and Microbiota Interplay in Pancreatic Cancer

**DOI:** 10.3390/cancers15143573

**Published:** 2023-07-11

**Authors:** Pratibha Malhotra, Ranjith Palanisamy, Jose A. Caparros-Martin, Marco Falasca

**Affiliations:** 1 Metabolic Signalling Group, Curtin Health Innovation Research Institute, Curtin Medical School, Curtin University, Perth, WA 6102, Australia; pratibha.malhorta@postgrad.curtin.edu.au (P.M.); ranjith.palanisamy@telethonkids.org.au (R.P.); 2 Wal-Yan Respiratory Research Centre, Telethon Kids Institute, Perth, WA 6009, Australia; jose.caparros-martin@telethonkids.org.au

**Keywords:** pancreatic cancer, microbiome, bile acids, pancreatic ductal adenocarcinoma, biomarkers

## Abstract

**Simple Summary:**

The gut microbiota is involved in homeostasis but can facilitate the insurgence of diseases including pancreatic cancer when altered. These gut microbes modulate the metabolism of bile acids, which are found to be abnormal in pancreatic cancer and diseases considered risk factors for it. Therefore, changes in the functional state of the gut microbiota may result in bile acid alterations, which eventually could promote cancer development. A better understanding of contribution of the gut microbiota in pancreatic cancer development would guide us to new strategies for early diagnosis and opportunities to improve a patient’s response to therapy. This review examines the current knowledge on gut microbiota and bile acid interrelation and their relationships with pancreatic cancer.

**Abstract:**

Evidence suggests the involvement of the microbiota, including oral, intra-tumoral and gut, in pancreatic cancer progression and response to therapy. The gut microbiota modulates the bile acid pool and is associated with maintaining host physiology. Studies have shown that the bile acid/gut microbiota axis is dysregulated in pancreatic cancer. Bile acid receptor expression and bile acid levels are dysregulated in pancreatic cancer as well. Studies have also shown that bile acids can cause pancreatic cell injury and facilitate cancer cell proliferation. The microbiota and its metabolites, including bile acids, are also altered in other conditions considered risk factors for pancreatic cancer development and can alter responses to chemotherapeutic treatments, thus affecting patient outcomes. Altogether, these findings suggest that the gut microbial and/or bile acid profiles could also serve as biomarkers for pancreatic cancer detection. This review will discuss the current knowledge on the interaction between gut microbiota interaction and bile acid metabolism in pancreatic cancer.

## 1. Introduction

Pancreatic cancer (PC) is a virtually incurable invasive cancer with rising global incidences and poor outcomes. According to Globocan 2020, the number of cases parallels the mortality rate [1]. The incidence rate varies across countries, with a generally increasing trend in developed nations compared to others [2]. PC can arise from both the endocrine and exocrine pancreas. Originating from exocrine tissue, pancreatic ductal adenocarcinoma (PDAC) is the most common form of PC, representing 90% of the cases [3]. 

Current therapeutic modalities for PC include surgery, radiotherapy, chemotherapy and palliative treatment [4]. Unfortunately, many PC patients suffer a relapse, and although undergoing potentially radical therapy, the overall 5-year patient survival rate is around 11% [5]. Thus, despite our extensive research efforts and increasing understanding of PC, effective therapies are a daunting challenge. These challenges include recognising and screening risk populations, identifying novel biomarkers for early detection, and improving therapies to overcome resistance to current treatment modalities and improve overall survival in PC patients [4]. 

The risk factors for PC can be stratified into modifiable and non-modifiable. The modifiable PC risk factors include smoking, obesity, alcohol consumption and dietary factors. On the other hand, age, chronic pancreatitis, gallstones, diabetes, blood group, ethnicity, genetics, and family history are classified as non-modifiable risk factors [2,3]. 

Recent evidence suggests the role of bile acids (BAs) and gut microbiota in PC. The altered BA pool has been associated with several disease states, including inflammatory bowel disease [6,7], metabolic syndrome [8], *Clostridium difficile* infection [9] and cancer [10,11], including PC. Interestingly, about 60% of PC arises from the pancreatic head close to the biliary tract, suggesting a potential involvement of BA in PC [12]. 

The gut microbial metabolism modulates the BA pool structure and is inherently associated with host physiology. Conversely, the size and composition of the BA pool are linked to the gut microbiome community and its composition [13]. Hence, changes in gut microbial functionalities may likely result in variations in the BA pool [14]. The microbiome supports nutritional and hormonal homeostasis, aids inflammation modulation, detoxifies compounds, and provides bacterial metabolites with metabolic modulating effects [15]. Diet, antibiotics, drugs, environmental stressors, exercise/lifestyle, and gastric surgery are known to modulate the microbiome. Other factors, such as geography, ethnicity, host genetics, age and gender, also contribute to the high interindividual variation in the microbiota observed in healthy individuals [16].

Recent studies have highlighted the crucial role of the microbiome in gastrointestinal cancers, including liver, colorectal and PC. Several microbial alterations exist in PC patients as opposed to healthy groups at several locations, including oral, gastrointestinal, and pancreatic tissues. A growing body of evidence suggests the implications of these microbes in PC predisposition, occurrence, progression and therapeutic efficacy [17]. While the mechanisms through which microbiota and BAs affect PC are being investigated in other cancers and diseases, their interactions in PC also need close attention. This review will focus on the potential influence of BA and gut microbiota on PC. 

## 2. Overview of Bile Acid/Host/Microbiota Interactions

**Figure 1:**
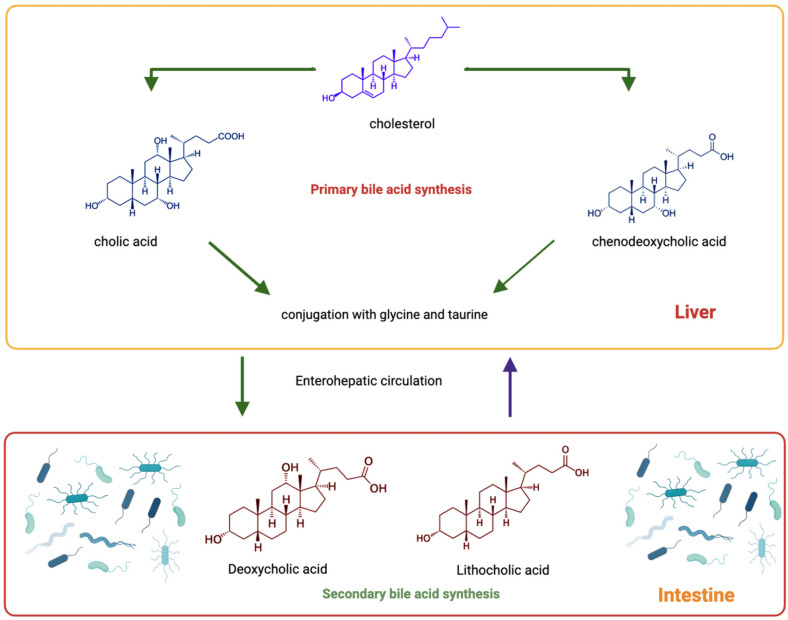
Bile acid synthesis. The liver synthesises two primary bile acids, i.e., cholic acid (CA) and chenodeoxycholic acid (CDCA). These bile acids are conjugated with glycine and taurine and undergo enterohepatic circulation. The bile acids that enter the large intestine are converted to secondary bile acids by the gut microbiota. Deoxycholic acid (DCA) and lithocholic acid (LCA) are the two main secondary bile acids produced in the human body. Some of these secondary bile acids are absorbed and conjugated in the liver.

The liver produces bile, a biological fluid composed of BA, cholesterol, electrolytes, phospholipids, bilirubin, and water [18]. BAs are amphipathic steroidal molecules synthesised from cholesterol in the liver. The synthesis of BA from cholesterol is a multienzyme step (Figure 1). The parenchymal cells (hepatocytes) encompass a set of 17 enzymes required for modifying the steroid core, removing side chains, and conjugating to taurine or glycine, resulting in the primary bile acids. Cholic acid (CA) and chenodeoxycholic acid (CDCA) are two major primary human BAs [19]. This biosynthetic reaction happens in the mitochondria, endoplasmic reticulum, peroxisomes, and cytoplasm. There are four different pathways for synthesising BAs, i.e., the classical, alternative, 25-hydroxylation and Yamasaki pathways. This cholesterol synthesis offers BA detergent-like properties that are crucial for physiological functions such as hepatic transformation and absorption of fat-soluble vitamins and dietary lipids. The chemical diversity of the BA pool is additionally expanded by the intestinal microbiota generating secondary BAs. The two major secondary BAs in humans are deoxycholic acid (DCA), produced from CA, and lithocholic acid (LCA) from CDCA [20]. A comprehensive explanation of the BA synthesis cascade is beyond the scope of this review and has been extensively presented elsewhere [21,22,23].

### 2.1. Bile Acid Synthesis and the Liver

The classical or neutral pathway is the most important biosynthetic pathway, producing 90% of BAs. The cholesterol 7 alpha-hydroxylase (CYP7A1) is the rate-limiting enzyme in this pathway, being involved in the catalytic hydroxylation of cholesterol, yielding 7α-hydroxy cholesterol [24]. The physiological importance of CYP7A1 is evident from the phenotype changes in the CYP7A1-deficient mice displaying abnormal lipid excretion, behavioural irregularities and skin pathologies [25]. The acidic or alternative pathway involves the conversion of C27 BAs and oxysterols produced in different cell types, which are circulated and transformed into BAs. Less than 10% of the total BA pool is synthesised using this pathway. While the alternative pathway is predominant during childhood, the classical pathway contributes significantly to the BA pool later in life [26]. 

Before BA secretion into the bile canalicular lumen, the side chain of primary BAs is conjugated with glycine or taurine by bile acid coenzyme A and amino acid N-acyltransferase (BAAT) to increase their solubility [27]. The physiological glycine-to-taurine conjugation ratio in human BAs is 3:1, which can be altered in disease. For instance, the percentage of taurine conjugates is elevated in cholestatic liver disease but lower in situations associated with a requirement to increase conjugation, such as BA sequestrant treatments and external biliary drainage [28]. This conjugation process lowers the pKa of BA, rendering them ionised at a physiological pH. In addition to glycine and taurine, BAs can undergo other modifications, such as sulfation and glucuronide conjugation [28].

### 2.2. Biotransformation of Primary Bile Acids

The gastrointestinal microbial population is a natural ecosystem encompassing 10^14^ bacteria. These microorganisms contribute to 99% of the functional genes involved in multiple regulatory roles [29]. Over 90% of bacteria in the human colon belong to Firmicutes and Bacteroidetes phyla. Other phyla, including Actinobacteria, Verrucomicrobia, Proteobacteria and Fusobacteria, contribute to gut diversity. The most prevalent identified genera include *Bacteroides*, *Propionibacterium*, *Ruminococcus*, *Lactobacillus*, *Bifidobacterium*, *Streptococcus*, *Eubacterium*, *Peptostreptococcus*, *Clostridium* and *Methanobrevibacter* [11].

Some of these intestinal bacteria contribute to the transformation of host-synthesised primary BAs to secondary BAs, thus altering the size and composition of the BA pool. Given their antimicrobial properties, BAs can, in turn, modulate the gut microbiota composition. Therefore, the BA pool is a synergic readout between the host and the gut flora [19]. The modification steps through which the intestinal flora can alter the BA composition and synthesise the secondary BAs involve deconjugation, dehydroxylation, oxidation, epimerisation, desulfation, unsaturation and esterification, thus generating a BA pool of considerable structural diversity [19,30]. 

The deconjugation step by the intestinal bacteria generates free BAs by hydrolysing glycine or taurine conjugates. This step is catalysed by the bile acid hydrolase (BSH) enzyme widely expressed in *Bifidobacterium*, *Brucella*, *Bacteroides*, *Clostridium*, *Lactobacillus*, *Stenotrophomonas*, *Listeria* and *Enterococcus* [31,32]. The BSH enzyme activity is an adaptation step for protecting against toxic BA conjugates. These free BAs are then available for subsequent bacteria-mediated transformations. One of the critical transformations mediated by catalysis by the bacterial 7α/β-dehydroxylase found in *Bacteroides*, *Lactobacillus*, *Clostridium*, *Listeria*, *Enterococcus* and *Bifidobacterium* is dehydroxylation. The enzyme converts primary BAs into secondary BAs, DCA and LCA [33,34]. The association of LCA and DCA with gallstones, obesity (risk factors for PC), and colon and liver pathology suggests that dehydroxylation is a physiologically important biotransformation pathway in the human intestine [33,35]. Deconjugation and dehydroxylation increase the pKa and the hydrophobicity of bile acids, thus improving colon absorption and subsequently facilitating their recovery [30].

The hydroxysteroid dehydrogenase (HSDH) enzyme in the bacteria can catalyse the reversible oxidation of primary and secondary bile acids, which can undergo epimerisation. This enzyme is reported in the major phyla Actinobacteria, Firmicutes, Proteobacteria and Bacteroidetes [30,33]. Epimerisation is considered a microbial adaptation system as it yields less toxic, hydrophilic iso-bile acids to augment microbial resistance in a competitive environment [36,37]. These iso-bile acids can also modulate gut microbial community structure and host metabolism. For example, a study reported iso-DCA favouring *Bacteroides* genus growth associated with obesity [36,38]. Desulfation of BAs by bile acid desulfatase activity makes them more hydrophobic and aids their efficient absorption, suggesting that bacteria with desulfatase activity can modulate enterohepatic circulation and increase the BA half-life. Studies have shown that desulfated bile acids are more toxic than sulfated ones and could play a role in hepatobiliary toxicity [30,39].

### 2.3. Enterohepatic Circulation Dynamics

**Figure 2:**
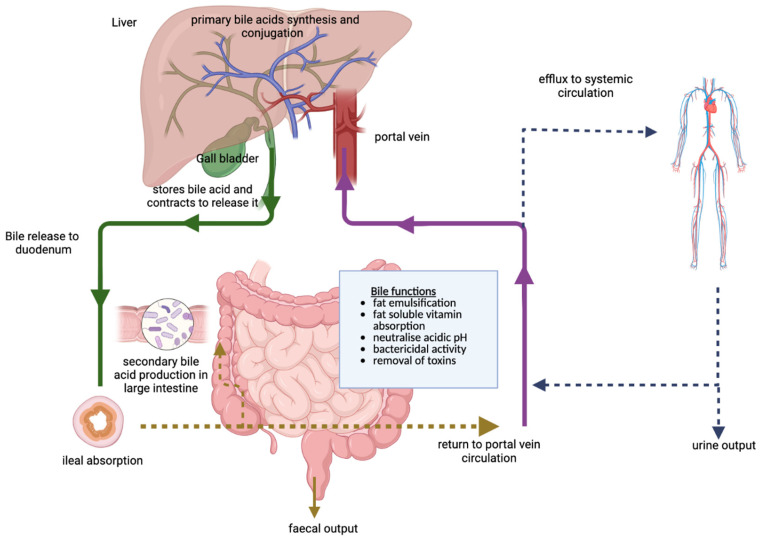
Bile acid circulation. Primary bile acids, i.e., cholic acid (CA) and chenodeoxycholic acid (CDCA), are synthesised from cholesterol in the liver. These bile acids are conjugated with glycine and taurine and stored in the gall bladder. The gall bladder contracts (via CCK), and the bile acids are released upon meal consumption. Most of the bile acid is absorbed from the ileum and returned to the liver via the portal vein (95%). During enterohepatic circulation, a small amount of bile can escape into the systemic circulation and form part of a circulatory bile acid pool. The unabsorbed bile acids in the large intestine are converted to secondary bile acids. These secondary bile acids are absorbed and added to the bile acid pool. About 5% of these are released as faecal output.

The total BA pool is ~1.5–4 g and is recycled between 4 and 14 times daily. The BA pool recovery rate of enterohepatic circulation is 95%, with only 5% (0.2–0.6 g per day) moving through the large intestine and contributing to the faecal loss. Thus, ~500 mg of BAs is newly synthesised in adults, representing 50% of the cholesterol turnover [20,40]. In humans, >90% of the total BA pool comprises CA, DCA and CDCA, which are well conserved and cycle through enterohepatic circulation (Figure 2).

Currently, it is unclear how the BA metabolism is physiologically regulated [41]. However, there are three independent perspectives on how bile acid metabolism is regulated in our bodies [41]. The first and main viewpoint is that the body maintains the BA pool at a certain level and compensates for any intestinal loss using de novo synthesis. The second perspective is that the BAs can activate the farnesoid-X-receptor (FXR) in the intestine, and the liver regulates the BA synthesis via a negative feedback inhibition loop [42]. The BA-FXR interaction is vital in glucose and lipid metabolism, inflammation, liver renewal and protein synthesis, as discussed elsewhere [43]. Different BAs bind to FXR with varying affinities. Finally, a diurnal variation in BA synthesis has been proposed [44]; however, the mechanism for BA synthesis reactivation or inhibition at night remains unknown [41]. Bilirubin is an important component of bile and acts as an antioxidant. It can form a part of the enterohepatic circulation and be actively secreted by hepatocytes [45]. 

The stored bile acids are released into the duodenum via cholecystokinin (CCK), which binds to the CCK receptor in the gall bladder and induces its contraction for emptying [46]. The gall bladder’s contraction duration and emptying rate can vary depending upon the CCK production capacity by the duodenum, meal size and composition and the gall bladder muscle response to CCK receptor stimulation. The bile facilitates fat emulsification and transit through the intestine to the terminal ileum to be reabsorbed. They are then reabsorbed actively in the distal ileum and passively in the small intestine and colon [41].

These reabsorbed BAs are returned to the liver via the enterohepatic cycle, where hepatocytes take them up and then are re-secreted. Active ileal bile acid absorption begins with sodium-dependent bile acid transport (ASBT) and protein-mediated enterocyte uptake, followed by intestinal bile acid-binding protein (i-BABP) controlled intercellular transport, and finally, organic solute transporting dimer (OSTα/β)-facilitated secretion into portal blood. The sodium-dependent taurocholate cotransporting polypeptide (NTCP) controls this active process. During the enterohepatic circulation, a small number of BAs can get shunted into the systemic circulation/peripheral bloodstream, allowing BA signalling in other tissues. In the large intestine, unabsorbed BAs can serve as substrates for microbiota, which can metabolise them to secondary bile acids, as discussed briefly in Section 2.2 [41]. Once reabsorbed and in circulation, these secondary BAs can be conjugated (similar to primary BA) and added to the BA pool. In addition, the microbes can modify both DCA and LCA to yield other secondary BAs. More than 50 distinct microbiome-derived secondary BAs are found in human faeces, with LCA and DCA being the predominant ones [29].

### 2.4. Gut Microbiota/Bile Acids in Host Physiology and Disease

The bidirectional, mutually beneficial dynamic interaction between the BAs and the gut microbiota is critical to maintaining normal homeostatic host physiology. The gut microbiota enriches the bile acid diversity and regulates their synthesis as well as transportation. These BAs, in turn, positively or negatively influence intestinal flora [11].

It is also known that BAs can exert anti-microbial effects on gut flora directly or through FXRα, which is involved in the intestinal mucosal defence [11], thus putting the gut through a fitness test resulting in a shift in structure. Gram-positive microbes are shown to be more sensitive to BAs than Gram-negative [47]. However, the BA resistance of Gram-negative species is less characterised. Compared to conjugated BAs, unconjugated BAs have stronger anti-microbial activity [48,49]. Furthermore, BA deficiency can increase pathogenic bacteria’s growth, thus increasing the risk for translocation, hydrophobicity, membrane damage, and inflammation [50]. Generally, an outgrowth of Gram-negative bacteria results from the drop in the BA pool.

On the contrary, with the increased BA levels, there is an observed growth of Gram-positive Firmicutes, including those with the ability to dehydroxylate BAs thus, promoting the production of secondary BAs [29]. The changes in the gut microbiota/bile acid profile can alter the host metabolic phenotype and can lead to several metabolic disorders. As aforementioned, microbiome dysbiosis is a pathophysiological feature of PC patients and will be discussed in the next section.

## 3. Microbiome Dysbiosis in Pancreatic Cancer

It is now established that the pancreas is a non-sterile organ and harbours its own microbiota [51,52]. A healthy pancreas has an essential role in gut microbiota management, and in turn, the gut microbiota plays a key role in regulating pancreatic function [53].

The pancreas secretes antimicrobial peptides in its pancreatic juice. Ahuja et al. reported that pancreatic acinar cells secreted antimicrobials, which were essential in shaping the gut microbiome, further influencing gut innate immunity, barrier function, and survival. Despite eliciting a strong gut innate immune response, the knockdown of mouse pancreatic acinar cell *Orai1* gene, encoding the **calcium release-activated calcium channel protein 1**, resulted in high mortality with severe intestinal bacterial outgrowth and dysbiosis. Furthermore, these *Orai1*-deficient mice had decreased levels of a major antimicrobial peptide, cathelicidin-related anti-microbial peptide (CRAMP), secreted by the pancreas [54]. Another study in mice associated CRAMP deficiency with increased inflammation and pancreatic injury [55]. These studies suggest that pancreatic cell injury may be associated with gut microbial dysbiosis. In addition, an increased gut bacterial burden has been reported in pancreatitis [56]. The translocation of bacteria from the gut into the pancreas is also associated with inflammation [57].

Research in this field points towards an intricate relationship between the microbiome and PC. Evidence has pointed towards the association of PC development and progression with the oral, gut and intratumor microbiomes, which has been elegantly discussed elsewhere and is beyond the scope of this manuscript [17,57,58,59,60,61,62,63].

### Microbial Metabolites in Pancreatic Cancer

The gut microbiome encompasses abundant species with large metabolic diversity. Microbiome-derived metabolites have emerged as essential factors in arbitrating the effects of the commensal microbiome on host physiology both locally and systemically [64]. Owing to microbiome dysbiosis, gut-derived bacterial metabolites can influence tumour progression [61]. Dysregulation of bacterial metabolism has been reported in PC [52,61,65]. 

Short-chain fatty acids are derived from the bacterial fermentation of non-digestible carbohydrates. Bacteroidetes primarily produce acetate and propionate, while butyrate is produced by Firmicutes [66,67]. Studies have suggested the protective effects of acetate [68], butyrate [69], butyrate conjugate hyaluronic acid [70] and other SCFAs such as valproic acid [71] in PC. Altering these SCFA-producing bacteria in PC suggests suppressing these protective effects [61]. Furthermore, lipopolysaccharide (LPS)-producing bacteria are commonly observed to be increased in PC patients [65]. LPS can elicit an immune response through toll-like receptors 2 (TLR2) and TLR4 [72,73]. Both these receptors and TLR9 are involved in PC development [74,75], thus indicating a potential role for LPS-actuated TLR signalling in PC.

Polyamine metabolism is also dysregulated in PC [76]. Compared to other mammalian tissues, the healthy pancreas has the highest amount of spermidine, a native polyamine [77,78,79]. PC cells were reported to have increased polyamine levels and import activity [76]. Mendez et al. reported microbial dysbiosis and increased circulating polyamine levels in KPC mice and PDAC patients [80]. Bacteria play an important role in tryptophan metabolism to produce indole derivatives [61]. The tryptophan metabolic enzyme indoleamine 2,3-dioxygenase has been noted in PC tumour cells, while the normal cells tested negative [81]. This study indicates that this enzyme might be involved in PC progression. However, more research is required to establish a positive correlation. 

As aforementioned, BAs are important microbial metabolites. The following section will discuss their role in PC progression and the BA/microbiota axis.

## 4. Bile Acids and Pancreas

Several PC risks factors, such as pancreaticobiliary maljunction, gallstones and pancreatitis, share BA metabolism dysbiosis and BA reflux as the common pathological feature [12].

BAs can interact with the pancreas under pathophysiological conditions through two pathways, i.e., systemic circulation and BA reflux into the pancreas [12,82]. A biliopancreatic reflux study reported BA reflux in the pancreas in six patients. They found that the reflux could be extensive enough to reach the tail of the pancreas [83]. Furthermore, bile duct ligation and pancreatic duct ligation resulted in the increased severity of pancreatitis, suggesting that systemically circulating BAs interact with the pancreas and can exacerbate the condition [84]. Therefore, the disease-exacerbating effect of BAs would not only require reflux into the pancreatic duct but could be elicited by serum or interstitial BAs in jaundice patients [85]. In addition, systemically circulating BAs also contributed to organ failure in pancreatitis patients [86]. Furthermore, a recent study in mice suggested a strong association between chronic pancreatitis and gut microbiota [87]. This indicates that the gut microbiota/bile acid interface influences pancreatic tissue pathology and needs closer attention.

### 4.1. Bile Acid Levels Are Dysregulated in Pancreatic Cancer

Studies have associated high physiological BA concentrations with gastrointestinal cancers [88,89,90]. Biliary obstruction is present in 64–77% of the PC tumours arising from the ampulla region [91,92,93]. Jaundice (hyperbilirubinemia) presentation may suggest advanced PC stages. Obstructive jaundice could be mild or severe, and its degree correlates with altered liver function [93]. Serum BA levels are elevated in obstructive jaundice [94]. Currently, circulating total BAs can be used as diagnostic markers in hepatobiliary diseases [95,96]. BA dysregulation is also reported in pancreatitis [86,97]. An investigation showed drastically decreased duodenal BA levels in severe chronic pancreatitis patients [97]. Lower intestinal BA levels have been shown in pancreatic insufficiency cases. This is attributed to the low pH BA precipitation, notably the glycol-conjugated BAs [98,99,100,101]. A recent research investigation demonstrated an association between higher total BA and poor prognosis in acute pancreatitis patients [86]. 

Research investigation in PC has demonstrated alternated BA levels in patients [102,103,104]. Elevated serum BA levels were reported in PDAC patients with and without obstructive jaundice (higher in patients with PDAC + obstructive jaundice) compared to healthy controls. In particular, the authors found higher concentrations of GCA, GCDCA, TCA and TCDCA [103]. Furthermore, another research group reported higher BA levels in serum and pancreatic juice [105]. The presence of BA in pancreatic tissues and pancreatic duct-derived cells was also reported [104]. Rees et al. compared the common bile duct BA composition in a pancreatic adenocarcinoma and benign group. The study reported that patients with PC tended to have elevated unconjugated BA levels. However, the lower patient number could limit the significant difference in unconjugated BA levels. Further, the authors observed a significant increase in CA in cancer vs. benign patients. The collected pancreatic fluid did not contain BAs [102]. Additionally, PC had higher CYP7A1 expression or total BA levels compared to normal cells, thus suggesting the presence of acidic pathway-mediated BA biosynthesis in the pancreas, particularly in PC [104]. In a recent research investigation, Wang et al. reported 18 differentially regulated metabolites in serum of PC and liver metastasis nude mouse model. The most notable differences were observed in BA levels, particularly TCA and CDCA, prostaglandin E2, glycine, guanosine monophosphate, vitamin D, and inosine. These findings along with the relevant enriched pathways in the Kyoto Encyclopaedia of Genes and Genome (KEGG) as well as a set of human metabolome database (HMDB) are predicted to assist early detection and improve prognosis in PC patients with liver metastasis [106].

The mechanism responsible for differential BA levels in the common bile duct in the two groups is uncertain. One possible explanation for the increase in unconjugated BAs in pancreatic adenoma patients could be the presence of hydroxylase-producing bacteria around the common bile duct. Another alternative explanation stems from the research on common bile duct stones. Sandstad et al. showed that stones in the common bile duct could obstruct the bile flow into the duodenum, resulting in bile stasis associated with the growth of bacteria [107]. In addition, bile duct obstruction can also result from pancreatic head tumours [102].

### 4.2. Bile Acid Receptor Expression and Pancreatic Cancer

BAs activate different signalling pathways in cancer aetiology (9). Along with FXR, BAs interact with the pregnane X receptor (PXR), liver X receptor (LXR), constitutive androstane receptor (CAR), vitamin D receptor (VDR), RAR-related orphan receptor gamma (RORγT) group H member, G protein bile acid receptor1 (GPbar1/TGR5), vascular endothelial growth factor receptor (VEGF receptors), formyl peptide receptor 1 (FMLP) and sphingosine -1-phosphate receptor 2 (S1PR2) (Table 1).

These receptors are expressed at various sites, including the gastrointestinal tract, myeloid cells, heart, and central nervous system. Different BAs interact with these receptors with different binding affinities. These BAs influence cell proliferation and apoptosis, carbohydrate lipid and energy metabolism, liver regeneration, heat adjustment and homeostasis through these receptors [126].

FXR receptor expression has been reported in several cancers, including PC. Lee JY et al. reported FXR to be highly expressed in five PC cell lines and PDAC specimens, suggesting its role in PC progression. The study also reported a positive correlation of FXR expression with lymph node metastasis, cell proliferation, migration, and invasion [108]. Additionally, Chen et al. found increased FXR expression in PDAC patients compared to normal, benign or precancerous tissue [109]. Increased BA levels and higher FXR expression confirmed higher activity in the PC tumours [105]. Furthermore, positive FXR levels were associated with cancer development and poor prognosis [109]. However, in another study, higher FXR expression in PC correlated with more prolonged survival and a less aggressive phenotype [110], indicating the conflicting role of FXR in PC.

TGR5 expression levels have been associated with gastrointestinal cancer, such as oesophageal cancer [127,128]. This receptor is expressed in the pancreas and plays an essential role in glucose metabolism [129,130,131,132]. Zhao et al. found increased expression of TGR5 in PC tissue specimens compared to healthy tissues. Furthermore, a higher receptor expression is correlated with tumour grade and lymph node invasion, suggesting this receptor’s pro-tumorigenic potential [111]. In addition, mice deficient in this receptor displayed milder pancreatitis upon exposure to taurolithocholic acid-3 sulfates (TLCS) [112]. However, how TGR5 contributes to poor prognosis needs to be investigated. TGR5 suppresses cell proliferation and migration in liver cancer [133], suggesting that the receptor might play different roles in different cancers.

Under physiological conditions, the PXR expression was much lower or negligible in the pancreas than in the liver [134]. However, in PDAC cell lines, Noll et al. reported increased expression of PXR comparable to that of the liver, suggesting its role in carcinogenesis. The authors also noted that the upregulated PXR gene with its transcriptional target CYP3A5 contributes to chemoresistance in PDAC [135]. Interestingly, Koutsounas et al. showed that the overexpression of PXR and its associated receptors had favourable outcomes in PDAC patients [113]. The results of these two studies indicate the need for more research on the role of PXR in PC. More recently, Oladimeji et al. reported N-alpha-acetyltransferase (NAA10) as a transcriptional factor contributing to regulating PXR by screening PC cell lines with an elevated PXR using the transcriptional factor siRNA library [136].

The VDR has been detected in different normal and cancer tissues. In the pancreas, increased expression of VDR has been reported in PC cells and tumour tissue compared to normal tissue [114,115,116]. Interestingly, VDR gene variations have been associated with PC [116,137,138]. Sherman et al. found that VDR is expressed in pancreatic tumour stroma and acts as a transcriptional regulator for pancreatic stellate cells (PSCs). The activation of stromal VDR overcame chemotherapeutic resistance and increased survival in combination with gemcitabine [117].

Similarly, another recent study showed that a combination of VDR and gemcitabine enhanced PC therapy through modulation of the tumour microenvironment [139]. VDRs were shown to favourably modulate tumour-stroma crosstalk by decreasing the release of exosomal oncogenic miRNA (miR-10a-5p) in PC [118]. This receptor has been implicated in protective desmoplasia [119], repressing PC cell stemness [120], as a prognostic factor and therapeutic target [121], as well as a determinant of survival [122].

The LXRs have been recognised to control cell growth in normal and cancer tissues. The LXRβ was abundantly expressed in PDAC tissues [123]. The LXR/RXR system components were also reportedly enriched in the serum of PC patients [124]. In addition, treatment with LXR agonists is reported to have anti-proliferative effects [125], disrupt glutamine metabolism and actuate oxidative stress in PC cells [140]. Recently, another new molecule, GAC0003A4 (3A4), has been demonstrated to impair phospholipid and cholesterol metabolism and concurrently induce cell death in PC cells [141].

Sphingosine 1 phosphate (S1P) plays an essential role in PC cell proliferation and migration through its receptors, i.e., S1PRs [142]. The S1PRs are expressed differently in malignant and benign tissues [143]. As mentioned above, one of the receptors, S1PR2, can interact with BAs. It is known that S1PR2 participates in pancreatic development, regulating lineage allocation and cell specification. S1PR2 is also known to stabilise the yes-associated protein (YAP) [144], which is overexpressed in PC and is recognised as a prognostic biomarker [145], suggesting an underlying role of S1PR2 in PC. Recently, Yang et al. reported TCA arbitrated S1PR2 to ERK signalling activation in PC cells. The investigators also found S1PR2/ERK to be a critical intracellular signal, facilitating gemcitabine insensitivity [104].

Gut microbiota can modulate BA receptors through secondary BAs [13] or directly [146]. In PDAC cells, DCA induced STAT3 and EGFR signalling by binding to TGR5 [147]. Therefore, targeting the BA receptors can be a potential intervention strategy; however, more investigations need to be undertaken in PC. 

### 4.3. Bile Acids Can Induce Pancreatic Injury

Research suggests that BAs can interact with pancreatic cells and induce injury through different pathways [148].

#### 4.3.1. Pancreatic Acinar Cells

BA’s detergent and non-detergent properties can contribute to BA-induced pancreatic acinar cell injury. The methods through which BA detergent properties induce acinar cell injury include intracellular calcium increase [149] and mitochondrial membrane depolarisation, resulting in consequent intracellular depletion of adenosine triphosphate (ATP) [148]. For instance, low concentrations of TLCS induced global calcium oscillations in mouse pancreatic acinar cells. Local and global calcium oscillations were produced by taurocholate (TC) as well as taurodeoxycholate (TDC) but at higher concentrations than TLCS [149]. TLCS and TDC-acid (TDCA), and TCDC have been reported to depolarise the mitochondrial membrane [150]. TLCS has also been shown to decrease the mitochondrial and cytosolic ATP levels in acinar cells [151]. The non-detergent BA mechanisms for acinar injury involve pathological initiation of zymogens through the actuation of phosphoinositol-3-kinase (PI3K) [152]. The cytosolic calcium concentration increase resulted in premature zymogen activation and acinar cell necrosis [153].

BA can cause acinar cell depolarisation by inducing a cationic current via non-selective channels [148]. At a low concentration, TLCS can induce these cationic currents in acinar cells [154]. BA interaction can also alter the chemokine expression in pancreatic acinar cells [155].

#### 4.3.2. Pancreatic Ductal Cells

Understanding the effects of BAs on ductal cells is of interest since these cells are the first to be exposed to BAs in the case of biliary reflux. In vivo studies have shown that different BAs, in addition to human bile, can increase the permeability of the pancreatic duct [156,157,158,159]. For example, increased main pancreatic duct permeability to HCO_3_^−^ and Cl^−^ was observed on exposure to BAs in mM concentrations [158,159]. Furthermore, different BAs have different effects on permeability, with the dihydroxy BAs exerting a more potent action than the trihydroxy BAs [156]. Though these in vivo investigations are highly significant, their translation to human disease is questionable. One reason is that these investigations used non-physiological BA concentrations that are unlikely to be present in the case of BA reflux into the pancreas.

Furthermore, these high concentrations cause excessive damage to the pancreatic ducts and acinar cells. In vitro studies have allowed the investigation of more pathologically relevant BA effects on pancreatic ductal cells [82]. For instance, Okolo et al. demonstrated that TDCA and TCDCA but not TCA resulted in a dose-dependent rise in K^+^ and Cl^−^ conduction in canine pancreatic ductal epithelial cells [160].

The effects of BA have also been investigated on inter/intralobular ducts in the pancreas. The primary function of these ductal cells is to wash digestive enzymes by releasing alkaline fluid rich in HCO_3_^−^ and neutralising acidic chyme in the intestine. In the past, studies have evaluated how BAs affect HCO_3_^−^ secretions [82]. Venglovecz et al. treated intra/inter lobular pancreatic ducts isolated from guinea pigs with conjugated and unconjugated BAs, i.e., chenodeoxycholate (CDC) and glycochenodeoxycholate (GCDC), respectively. The results of this study indicate that a low BA concentration could stimulate HCO_3_^−^ secretion, thus protecting the pancreas from toxic bile. However, higher concentrations could inhibit this secretion, thus contributing to pancreatic injury [161]. This indicates that BAs can have dose-dependent protective and harmful effects on ductal cells. 

#### 4.3.3. Pancreatic Stellate Cells

Pancreatic stellate cells (PSCs) are involved in developing the morphological characteristics of pancreatic injury and tissue fibrosis [162]. However, there are not enough data on how BAs affect PSCs. The NaT co-transporting polypeptide (NTCP) expression in the PSCs indicates the Na^+^-reliant BA uptake pathway. Ferdek et al. demonstrated the effects of BA in human cells (in vitro) and murine pancreatic lobules (ex vivo). BA treatment resulted in PSC necrosis.

Further treatment with sodium taurocholate and sodium cholate increased the cytosolic Ca^2+^ levels in PSCs more than in the proximal acinar cells. On the contrary, TLCS, known to elicit acinar cell calcium oscillations, had little effect on PSCs. Although acinar and PSCs are close to the pancreatic lobules, the differences in response to BA indicate that they display distinct sensitivities to pathophysiological stimuli [163]. Although this study highlights how BA affect PSCs and its role in pancreatic pathology, more studies are required to understand the BA effects on PSCs.

While some BAs are associated with pancreatic injury, their protective roles are also documented in the literature. For example, ursodeoxycholic acid (UDCA) [164,165] and taurourodeoxycholic acid (TUDCA) [166,167] have protective functions in acute biliary pancreatitis. In addition, TUDCA has been shown to reduce acinar cell injury and pancreatic inflammation [167]. These studies indicate that BA has a biphasic role in pancreatic injury.

### 4.4. Bile Acids in Pancreatic Cancer Pathogenesis

As aforementioned, obstructive jaundice is a common clinical manifestation of PC. Though surgery is the most effective option, applying a preoperative biliary decompression strategy to reduce surgical complications is still controversial [93]. Furthermore, there is still no consensus on whether PC’s reportedly elevated BA levels have harmful or protective functions. 

Research studies have indicated the pro-carcinogenic role of BAs in PC. Tucker et al. suggest the involvement of BA in PC pathogenesis via cyclooxygenase 2 (COX-2). The study demonstrated that both unconjugated and conjugated BAs could induce COX-2 and prostaglandin E2 in two PC cell lines. Due to hydrophobicity differences, the unconjugated BAs could induce COX-2 expression at a lower concentration than the conjugated [168]. DCA has been shown to induce cell cycle progression in PC cell lines by activation of the signal transducer and activator of transcription 3 (STAT3), mitogen-activated protein kinase (MAPK) and epidermal growth factor receptor (EGFR) signalling via the TGR5 receptor [147].

BAs can promote cancer progression through alterations in mucin expressions. Joshi et al. demonstrated that high BA levels could exacerbate PC tumorigenicity by upregulating mucin 4 (MUC4) via activation of the FXR/FAK/c-Jun axis [105]. Elevated expression levels of MUC4 in PC correlate with poor prognosis [169,170]. Downregulation of this mucin decreased cell growth in vitro and in vivo [171]. A more recent research study demonstrated that different BA treatments increased the proliferation, migration, invasion, adhesion and colony-forming ability of Capan-1 and BxPC-3 [103]. Furthermore, in terms of survival, compared to PC cell lines, BAs induced different effects on a normal pancreatic cell line, human pancreatic ductal epithelial cells (HPDEC). The authors report that BA decreased viability in HPDEC, which aligns with the studies showing BA can induce damage to normal PC cells, as discussed in the previous section. The different response could be due to the likeliness of BA to cause DNA damage rather than apoptosis in cancer cells. Since DNA damage is associated with frequent genetic alterations, this favours cancer progression. The authors also found that BA treatment of HPDEC resulted in the downregulation of MUC2 and upregulation of MUC20. Since MUC20 upregulation is known to support tumour development and MUC 2 is a tumour suppressor, this study’s results suggest that BA aids tumour progression under normal conditions [103]. Similar to Joshi et al. [105], this study also showed that BA treatments upregulated MUC4 in PC cell lines [103]. 

Evidence suggests the involvement of S1P signalling in BA-mediated PC progression. Sarkar et al. demonstrated that conjugated BAs could exacerbate metastatic PC via sphingosine 1 phosphate receptor 2 (S1PR2). The study showed that dose-dependent TCA treatment promoted AsPC-1 and Panc02-luc proliferation due to dominant S1PR2 but did not affect Panc-1, BxPC-3 and MIA PaCa-2, which expressed other S1P receptors [172]. However, the study was limited by using only one conjugated BA. Similar results have been shown in oesophageal adenocarcinoma [173]. Another recent investigation by Sarkar et al. showed the involvement of conjugated BAs in promoting PC progression via the S1PR2 receptor both in vitro and in vivo [174].

Some studies have demonstrated that BAs have protective roles in PC. For instance, Kim et al. demonstrated that UDCA could suppress stem cell formation and epithelial-mesenchymal transition in PC cells [175]. Lu et al. showed that BAs were cytotoxic on PC cell lines. Treatment with a bile-modified medium inhibited PANC-1 and MIA PaCa-2 cell proliferation and was reversible. The authors also reported that BA treatment altered PC cells’ morphology [176]. Similar inhibitory effects were observed by Wu et al. in PC. This study further suggested that elevated serum BA in jaundiced patients may impede PC progression [177]. For example, a recent study demonstrated that increased BA levels induce PC cell apoptosis through the ROS pathway [178]. Similar to these studies, a recent study demonstrated that patient (undergoing pancreaticoduodenectomy)-derived bile samples could decrease the peritoneal metastasis of Panc02 cells in vivo [179]. However, the observed effects are dependent on the BA concentration used. The inhibitory effects were observed at relatively low concentrations (<50 μM) [176,177].

In contrast, Gal et al. observed proliferative effects with relatively higher BA concentrations [103]. Similar dose-dependent responses have been reported by another study using CDCA in guinea pig pancreas [161]. These BA biphasic responses have also been documented in colon cells [180] and gastric cancers [181,182]. However, the explanation behind this phenomenon has yet to be discovered. In addition to BA concentration, interestingly, BAs display functional selectivity, for instance, in the case of UDCA. Therefore, more studies need to be undertaken to determine the involvement of BAs in PC and other cancers.

#### Bile Acids and Autophagy in Pancreatic Cancer

Autophagy is a sequential process for degrading long-lived proteins and cytoplasmic organelles. The process is regulated by the conjugation of phosphatidylethanolamine (PE) with LC3 (ubiquitin-like protein) via enzymatic steps catalysed by ATG3, ATG7, and ATG12-ATG5 complexes. It has important implications for cell survival during starvation periods and is also becoming recognised as a player in cellular homeostasis. In PC, autophagy is documented to have dual roles. While it is known to act as a tumour suppressor in the early stages, in the advanced PC stages, it supports survival and tumour growth during stress, including nutrient deprivation, hypoxia and chemotherapy [183]. The involvement of autophagy in PC and its regulation have been elegantly discussed elsewhere [184].

The activation of FXR can inhibit autophagy activation by downregulating the expression of genes involved in autophagy, such as ATG7, thereby implying a link between BA, their effect on FXR, and autophagy [185]. The FXR activation depends on the BA type [148,185]. For example, while conjugated BAs can activate FXR, CDCA can inhibit its signalling. Inflammation plays a significant role in pancreatic tumour development. Zhou et al. recently reported that BA contributes to pancreatitis by FXR activation in the acinar cell, which results in autophagy suppression, thereby prompting cell death, inflammation, and fibrosis in chronic pancreatitis [185]. The role of BA in acinar cell injury has already been mentioned in the previous section. The involvement of FXR in pancreatitis aligns well with its association with poor survival in PDAC. In contrast, a recent study reported that elevated FXR expression had a protective role in pancreatitis, as its activation resulted in autophagy restoration via OSGIN1 expression [186].

BAs can also influence PC progression through autophagy modulation. Knockdown of FXR receptor-suppressed AKT-mTOR pathway activation abolished its ligand-associated autophagic flux suppression and reinstated YAP degeneration [187]. Interestingly, a study demonstrated that pitavastatin and metformin synergistically inhibit PC progression through AMPK activation and PI3K/mTOR inhibition [188]. 

Further, research has shown that autophagy induction in PC due to hypoxia-associated ROS production leads to the breakdown of MUC4. This degradation supports the survival of stressed cells by providing them with the necessary metabolites [189]. Interestingly, BAs have been shown to promote PC progression through MUC4 expression [103,105,190].

A complicated interplay exists between lipid metabolism and autophagy [191]. Studies have shown that ferritinophagy or autophagic degradation of ferroportin-1 can increase free iron accumulation in cells, triggering Fenton chemistry-associated ROS production for ferroptosis in PDAC cells [192]. PC cells use ferritinophagy machinery to preserve a labile iron pool (LIP). The inhibition of autophagy can deplete LIP and result in mitochondrial function impairment. In a recent study, the authors showed that PC cells could compensate for the autophagy-inhibited associated LIP depletion by initiating paracrine signalling in neighbouring CAFs to trigger the ferroportin expression, resulting in therapeutic resistance [193].

Additionally, Dai et al. demonstrated that autophagy-dependent ferroptosis could release KRASG12D from PDAC cells. Their findings show that KRASG12D could promote fatty acid oxidation in cancer cells and macrophage polarisation to aid tumour progression [194].

### 4.5. Bile Acid/Gut Microbiome Axis in Pancreatic Cancer

It is already known that both BAs and gut microbiome interdependently modulate host gut homeostasis. A dysbiosis of BAs and/or microbiota in pathophysiology, such as cancer, alters this homeostasis. For example, gut microbial alterations can contribute to liver pathologies, mainly cholangiopathies, by altering BA composition and biliary immunity [195].

The gut microbes can translocate to and colonise the pancreatic duct and therefore are present in PC tumours [196,197]. These microorganisms can also translocate to the pancreas via bile. Studies have suggested the presence of microorganisms in bile. A study group reported that among the patients undergoing pancreaticoduodenectomy, 17% of patients without preoperative bile duct catheterisation (PBDC) had a positive bacterial culture, while 84% of the PBDC samples were positive for anaerobic and/or aerobic bacterial cultures. The group also reported the presence of occasional positive fungal culture [198,199]. In 21% of patients, the authors found similarities in the bacterial species at the surgical site (pancreaticobiliary surgery) and the bile fluid. Different bacteria can have different sensitivities to BA [198]. For instance, Enterococci are considered BA-resistant, which could explain their increased abundance in PC [197,200,201]. 

Studies have previously reported that infected bile is more harmful to pancreatic ductal cells [156,158,159]. A possible explanation could be the higher toxicity associated with bacterial deconjugation resulting in unconjugated BAs. This could further be influenced by the BA ionisation state and the pH [82]. A recent investigation investigated the effects of contaminated BA samples in PC. While BAs could decrease PC metastasis in murine models, the contaminated samples diminished this protective response.

Further, the authors reported modification of the anti-carcinogenic characteristics by incubating sterile bile with live *Streptococcus oralis* or *Enterococcus faecalis*. This change was not recorded in culture media following live bacterial incubation. This study indicated that bacteria could alter the cytotoxic properties of bile. As discussed in the previous sections, many studies have reported that different BAs can affect cell survival. This property of BAs is probably governed by the ability of the microbial population to deconjugate primary BAs. The authors found differences in the anti-carcinogenic properties of conjugated and unconjugated BAs at the same molar concentrations [179].

Although this study suggests that the ability of bacteria to alter BA properties can affect PC progression, major studies are required to understand whether this microbial contamination can alter clinical oncological outcomes [179]. Also, research is needed to understand which BA type and bacterial stain have a cooperative or a dominant effect in PC cancer biology. Therefore, there is a need to understand PC’s microbiome/BA axis in more depth.

A more recent study by Zhao et al. found that hypernedotoxemia and hyberbileacidemia play a role in PC development [187]. The authors found increased plasma LPS and bile acid levels in pancreatic cancer mouse models harbouring the KRAS mutation, indicating their link with PC progression. The research demonstrated that LPS injection or common duct ligation impaired autophagic flux, resulting in Yes-associated protein (YAP) accumulation. On the contrary, cholestyramine (resin) administration’s sequestration of endotoxins and bile acids promoted YAP degradation. On a cellular level, the study demonstrated that CDCA or LPS activates the AKT-mTOR pathway, increasing autophagic flux and accumulating yes-associated proteins (YAP) through FXR and TLR receptors. Knockdown of TLR and FXR receptors reduced the flux and promoted YAP degradation. YAP activation promotes PC progression and fibroblast activation, while its downregulation prevents fibrosis and tumour growth in PC. Therefore, the study proposes using cationic resins as an intervention strategy for PC [187].

## 5. Microbiota and Microbial Metabolites in PC Therapy

Most PC patients require a systemic therapeutic approach, whether it encompasses surgery followed by adjuvant, neoadjuvant or palliative therapy. Nevertheless, many patients develop resistance or toxicity to these drugs [202]. Consequently, these adverse events may necessitate drug dose reduction to make the treatment tolerable or suspension before the treatment completion date [203]. It is worth noting that the resulting microbial dysbiosis from PC can promote its pathogenesis and affect therapeutic outcomes. Microbiota and microbial metabolites, including BAs, can contribute to positive or negative therapeutic responses in PC. This section will focus on the interdependent bi-directional relationship between the microbiota, microbial metabolites, and BAs and PC therapeutic approaches.

### 5.1. PC Therapeutic Approaches Alter Microbiota

As mentioned previously, PC and its associated risk factors lead to host microbial dysregulation. However, surgical intervention and other therapeutic strategies can exacerbate this dysregulated state, resulting in adverse events [204,205].

#### 5.1.1. Surgery

Pancreatectomy is a complex PC surgical procedure associated with complications such as postoperative pancreatic fistula (POPF), varying in severity [206]. Previous research has shown that patients developing post-pancreatic surgery complications are not likely to undergo adjuvant therapy and consequently have a lower survival rate [207]. In addition, the microbial pathogens detected in the pancreatic fistula can govern the clinical outcomes of surgery [208]. Demir et al. concluded that multi-drug resistant microbes and Enterobacterales were frequently detected post-pancreatic surgery and were associated with severe complications, including pancreatic fistula in patients undergoing distal pancreatectomy [209]. These studies suggest that PC surgery could reshape the pancreatic microbiome. Schmitt et al. investigated the changes in microbial structure following pancreatic cancer surgery and its impact on the post-surgery course of the patients. The study did not observe dramatic changes in the patients’ alpha diversity and microbial richness. However, in the patient group presenting post-surgery complications, the microbial pattern indicated slight alterations. The authors reported that patients presenting with enrichment in Enterobacteriaceae, *Akkermansia* and Bacteroidales plus depletion of *Prevotella*, *Bacteroides* and Lachnospiraceae had a significantly higher risk of developing post-surgery complications [210].

#### 5.1.2. Chemo/Radiotherapy

Chemo or radiation therapy alone or in combination are therapeutic options for PC patients. These approaches have brought a slight improvement in the survival of PC patients [202].

Chemotherapeutic drugs can reshape the microbiota composition, altering the host response. Gemcitabine is the first line of treatment for PC patients with good performance status [202,211]. Its administration enriched pro-inflammatory flora in patient-derived xenograft PC mouse models. The authors reported that gemcitabine reduced Firmicutes and Bacteroidetes, while this downshift replaced increased Proteobacteria and Verrucomicrobia [212]. An increase in Proteobacteria and a reduction in Firmicutes are associated with intestinal inflammation and IBD, respectively [213,214]. Therefore, shifting the gut microbiota to a pro-inflammatory profile post-gemcitabine treatment may be accountable for gastrointestinal mucositis and chemotherapy-associated side effects. This alteration paradoxically increases inflammation, thus contributing to PC. Inflammation causes intestinal permeability, thus allowing bacteria to translocate to distal organs via the bloodstream [212].

Gemcitabine has been combined with other chemotherapeutic drugs, such as paclitaxel and erlotinib, for better outcomes. The combination of albumin-bound paclitaxel and gemcitabine is a category-one recommendation for PC patients. Florez et al. did not find bifidobacterial, lactic acid bacteria or other intestinal bacteria susceptible to paclitaxel [215]. However, Loman et al. reported that paclitaxel-treated mice had altered intestinal bacterial populations. They reported that paclitaxel treatment decreased butyrate-producing bacteria and increased levels of *Mucispirillum* in the colon, which may effectuate chemotherapy-induced neuro-inflammation [216]. In addition, chemotherapy combinations containing paclitaxel probably cause *Clostridium difficile* infection [217]. Administering the combination of gemcitabine with erlotinib, an epidermal growth factor receptor tyrosine kinase inhibitor, showed increased survival in PC patients. This combination strategy is another option for patients with advanced PC [218,219]. Although little is known about the interaction between the microbiome and erlotinib, two studies have reported that certain intestinal bacteria did not show susceptibility to erlotinib and alter intestinal tissue morphology. However, it is unknown whether erlotinib changed the gut microbiome structure [215,220]. The cumulative effect of the combinatorial administration of gemcitabine and erlotinib on the microbiome remains to be investigated. 

Another combinational chemotherapeutic drug, FOLFIRINOX (irinotecan, 5-fluorouracil, oxaliplatin and leucovorin), is considered a first-line therapy regimen for patients presenting with advanced and metastatic PC [202]. Oxaliplatin, a third-generation platinum-based drug, has been approved for use in FOLFIRINOX for PDAC treatment. Stojanovska et al. reported that oxaliplatin treatment alters the gut microbiota profile. They reported that oxaliplatin treatment enriched *Odoribacter* and *Prevotella2* but significantly reduced *Prevotella1* and *Parabacteroides* bacteria [221]. In addition, 5-fluorouracil (5-FU) treatment is reported to alter the microbial composition by enhancing *Staphylococcus* and *Clostridium* and reducing *Lactobacillus* and *Streptococcus*, resulting in decreased mucin secretion, a critical factor for the physiological defence mechanism of gastrointestinal mucosa [222,223]. Another study reported 5- FU changes in the microbial community composition compared to the control group [224].

Compared to gemcitabine, although FOLFIRINOX treatment improved overall survival outcomes in metastatic PDAC patients, the drug toxicity was also higher [225]. One of the agents used in FOLFIRINOX treatment is irinotecan (CPT-11), which occasionally results in toxicity, thus limiting the efficacy and regimen use. In addition, one of the typical clinical effects of irinotecan is delayed onset diarrhoea, thus suggesting that this drug can alter the gut microbiota profile [203]. Lin et al. demonstrated that irinotecan-based treatment altered intestinal microbiota in tumour-bearing rat models [226]. Rats treated with irinotecan showed significantly reduced overall microbial diversity with an increased abundance of Proteobacteria and *Fusobacterium*, which have been linked to a pro-inflammatory intestinal state [227]. More research is required to understand how irinotecan alters the gut microbial profile and its impact on overall survival outcomes in PC.

Radiotherapy is an important treatment modality for advanced PC patients [228]. Several studies have reported that radiotherapy can result in alterations in the microbiome [229,230,231]. Furthermore, the gut microbiome and radiotherapy interaction are known to be bidirectional, as radiotherapy-mediated microbiome disruptions can influence the therapeutic strategy’s effectiveness [232]. Nonetheless, little is known about how radiotherapy alters microbial composition in PC patients.

### 5.2. Microbiota Alters Host Response to Pancreatic Cancer Therapeutic Options

The microbiota has the potential to alter the pharmacotherapeutics of cancer treatment. Accumulating evidence has shown that the gut microbiota can contribute to the therapeutic efficacy of traditional chemotherapy through drug metabolism, biotransformation, and immune modulation [233,234].

Microbiota can promote pro-tumoral host responses by modulating the gene expression and metabolic activity of certain drugs. For example, *Mycoplasma hyorhinis* could compromise the cytostatic activity of gemcitabine via cytidine deaminase (CDD), resulting in rapid drug catabolism [235]. Geller et al. further showed that an isoform of CDD is expressed by Gammaproteobacteria, which can metabolise gemcitabine to a less active form, thus decreasing drug sensitivity. Furthermore, the group reported that out of 113 human PDAC samples, 76% tested positive for Gammaproteobacteria [236]. *Escherichia coli* could also decrease gemcitabine efficacy by accelerating drug metabolism [237].

*Mycoplasma hyorhinis* can also contribute to the catabolism of fluoropyrimidines, including 5-FU [238]. *Fusobacterium nucleatum* is known to decrease sensitivity to 5-FU in colorectal cancer [239]. Another study showed an association between *Fusobacterium nucleatum* abundance and chemoresistance in colorectal cancer patients administered 5-FU-based adjuvant chemotherapy post-radical surgery. The authors further showed the infection by *Fusobacterium nucleatum* decreased the chemosensitivity of CRC cells by inducing BIRC3 [240], an IAP family member, which inhibits apoptosis by inhibiting the caspase cascade [241]. Similar to 5-FU, *Fusobacterium nucleatum* has also been associated with oxaliplatin resistance [239].

Gellar et al. reported the involvement of *Pseudomonas aeruginosa*, *Klebsiella pneumoniae* and *Citrobacter freundii* in Oxaliplatin resistance [236]. However, the underlying mechanism of resistance is currently unknown. In addition to altering the host response, the microbiota is also involved in chemotherapy-associated side effects. For instance, oxaliplatin’s efficacy is limited by gastrointestinal toxicity and chemotherapy-induced peripheral neuropathy. A study demonstrated the role of gut microbiota in inducing oxaliplatin-associated mechanical hyperalgesia in germ-free mice [236].

In addition to chemoresistance, the gut microbiota can mediate chemotherapy-associated drug toxicity. Irinotecan, for example, exerts its anti-cancer effects after conversion to SN-38, its active metabolite by tissue carboxyl esterase. However, SN-38 can cause gastrointestinal toxicity. Before excretion in the gut, the liver can convert the active metabolite (SN-38) to the inactive form (SN-38-G) via UDP-glucuronosyltransferase (UDPGTs). However, microbiota from the Firmicutes phylum via β-glucuronidases can mainly convert the SN-38-G into SN-38 [242], thus potentially inducing toxicity. Interestingly, a higher dose of irinotecan in germ-free mice showed less gastrointestinal damage compared to conventional models [243].

A growing body of evidence supports the gut microbiota’s role in radioresistance [244,245]. However, this interdependent relationship remains to be profoundly explored in PC.

The microbiota can support the host’s response to therapy. For instance, the commensal bacteria can modulate the response to oxaliplatin via ROS production [246]. Further studies are required to understand whether the bacteria could modulate other cancer therapies based on ROS production. Therefore, it is important to remodel the microbiota to favour therapeutic efficacy and reduce drug toxicity effects.

### 5.3. Remodelling or Combining Microbiota Can Potentiate the Anti-Cancer Therapy Options

Studies have tried to reprogram the gut microbiota to potentiate the therapy response and reduce the associated side effects. Kawaguchi et al. determined whether gemcitabine resistance could be overcome by combining *Salmonella typhimurium* A1-R with gemcitabine in a patient-derived orthotopic xenograft model derived from 2 PC patients. This study showed that in combination, S. *typhimurium* A1-R potentiated gemcitabine and significantly reduced tumour growth. Furthermore, the study showed that combining the GEM + nab-paclitaxel was only effective in one model, while the GEM + S. *typhimurium* A1-R showed regression in both models [247]. *S. typhimurium* has been shown to independently reduce tumour weight and area with comparable efficacy to gemcitabine, 5-FU, and cisplatin [248]. 

Pre-administration of *Escherichia coli* Nissle 1917 (EcN) in mice ameliorated Irinotecan-associated weight loss and diarrhoea. Furthermore, the study showed that EcN decreased intestinal permeability in irinotecan-treated mice, suggesting its protective role in irinotecan-associated intestinal injury [249]. In addition, the supernatant of *Lactobacillus plantarum* improves chemosensitivity to 5-FU in colorectal cancer [250]. More investigations are required in this field in PC.

Remodelling microbiota using antibiotics, probiotics, prebiotics, and diet can help improve therapeutic outcomes in PC [245]. For example, studies have reported the association between antibiotic use and gemcitabine efficacy. Sunakawa et al. demonstrated that antibiotic treatment improved gemcitabine treatment efficacy in advanced PC patients [251]. Faecal microbiota transplant (FMT) is under active investigation in PC to show that mice transplanted with stool samples of long-term PC patient survivors had less tumour progression than those transplanted with samples from short-term PC survivors [200].

### 5.4. Microbial Metabolites in Pancreatic Cancer Therapy

Microbial metabolites contribute not only to PC development and progression but could also take part in chemotherapeutic resistance. For example, Kesh et al. investigated whether microbial metabolome influences the chemoresistance of PC tumours implanted in T2D mice. Their data showed the enrichment of microbial metabolites that offer protection against oxidative stress in the altered drug metabolism pathways of treated and untreated T2D models. In particular, the authors reported the augmentation of microbial metabolites menaquinol and queusosine, which can act as protectant metabolites against antioxidants and protect cells against ROS accumulation induced by chemotherapy. The authors further propose the possible contribution of the enriched microbial metabolic pathways in the T2D group in their therapy-resistant nature [252]. In line with this, a recent study has revealed a correlation between the enrichment of microbiota-associated tryptophan derivative indole-3-acetic acid (3-IAA) in PC patients and their response to chemotherapy [253].

On the contrary, microbial metabolites have also been trialled in combination with chemotherapeutic treatments in PC. Butyrate treatment attenuates the 5-FU-associated intestinal damage [254]. In combination, butyrate potentiated gemcitabine to induce PC cell apoptosis [255]. Valproic acid increased the therapeutic efficacy of 5-FU in PC cell lines, thus indicating a potentially promising tool in PC therapy [71]. Furthermore, a study suggests using polyamine inhibitor SBP-101 in combination with gemcitabine and nab-paclitaxel as a first-line treatment for patients presenting with metastatic PDAC [256]. Similar to these studies, oral intake of 3-IAA, dietary adjustments, and faecal microbiota transplantation can enhance the effectiveness of chemotherapy in a PDAC mouse model [253]. BAs also play an important role in chemotherapy and will be discussed in the next section.

#### 5.4.1. Therapy Alters Bile Acid Levels in Pancreatic Cancer

Chemotherapeutic treatments can alter the BA levels in cancer patients [257]. A study compared the serum lipid profile of mice treated with gemcitabine, butyrate, and gemcitabine + butyrate [255]. The authors reported that compared to the control, gemcitabine treatment except TDCA increased DCA, CDCA and TCA in mice serum [255]. Based on the results, the authors speculated whether the gemcitabine + butyrate combination could restore BA homeostasis in the mice [255]. A recent study demonstrated that an increase in total circulatory BA post-chemotherapy is associated with a faster recovery. The authors further report variability in the individual BA composition in patients [257]. However, the study detected GCDCA and GUDCA in all the cases analysed. GCDCA and UDCA are known to play an important role in reducing ER stress and detoxification [257,258,259].

#### 5.4.2. Bile Acids Affect Therapy Outcomes

BAs can also influence the host’s response to therapy. The results from the study conducted by Yang et al. demonstrate the involvement of ABCA8 (member of the ATP binding cassette transporter)-mediated efflux of TCA in regulating chemosensitivity in PC cells. The results further showed the role of the TCA-S1PR2-Erk pathway in inducing gemcitabine insensitivity associated with ABCA8 [104]. On the contrary, a recent study indicated that BA (TUDCA) supplements could improve recovery in mice from the 5-FU treatment [257].

It is currently not well established how intricate the bile acid/chemotherapy axis is in PC. Although we have discussed the involvement of BAs in pathogenesis, more investigations are required to understand the role of individual BAs in chemotherapy. In addition to BAs, chemotherapy drugs can further increase bilirubin (bile component) levels. Thus, chemotherapy-related hepatotoxicity is also one of the causes of hyperbilirubinemia [260]. Gemcitabine administration could result in increased bilirubin and transaminase levels. Idiosyncratic liver failure is also one of the uncommon reactions to gemcitabine [260].

The pharmacokinetic properties of chemotherapeutic treatments can be altered due to previously present liver dysfunction. Hyperbilirubinemia impacted paclitaxel clearance negatively in patients presenting with solid tumours [260]. Most trials exclude patients with abnormal serum liver tests, including high bilirubin levels. Thus, many patients are excluded from treatment with potential benefits [261]. Since jaundice is commonly reported in PC, the feasibility of chemotherapy in these patients is limited by the number of studies. Some studies recommend lowering chemotherapy doses in cancer patients with hyperbilirubinemia [262,263,264]. However, these studies are limited by small sample sizes and variable degrees of liver function impairment [261].

## 6. Conclusions and Future Directions

This manuscript evaluates the dynamics of the gut microbiota and its associated metabolites in PC. Given that BA metabolism plays a vital role in modulating host-microbiota interaction, we have mainly focused on these microbial metabolites. We discuss the gut microbiota/BAs alterations in PC, their role in modulating therapeutic efficacy and host response in PC therapy regimens.

The microbiota can also serve as markers for early PC diagnosis. Early PC detection remains an urgent unmet need. Currently, the available biomarker for clinical use, carbohydrate antigen 19-9 (CA19-9), suffers from limited specificity and sensitivity for PC [265]. Studies have proposed PDAC markers in the blood [266], tissue [267], and urine [268,269] with limited applicability. A recent study taxonomically profiles tumour biopsies, saliva and faecal samples in the PC population compared to the healthy control [270]. Faecal microbiota-based classifiers accurately predicted PC irrespective of the disease stage. The stool samples of PC patients were enriched in *Streptococcus*, *Veillonella* and *Akkermansia*. The authors also validate the comparative abundance of *Lactobacillus*, *Bacteroides* and *Bifidobacterium* in PC tumour tissue.

Interestingly, the authors did not find any saliva microbiome signature association with PC that has been previously reported in other studies (*P. gingivalis*, *S. thermophilus*, *Fusobacterium* spp.) [270]. However, it is important to recognise the technical aspects that remain to be addressed. The limited taxonomic resolution of 16S sequencing, experimental heterogeneity, and different analytical approaches could account for the different results observed [270]. For example, from the biomarker perspective, we aim to identify a rare microbial signature/genotype associated with the host phenotype (e.g., disease pathology). It is important to address the limitation due to overall microbial and metabolomic diversity. The level of a particular microbial metabolite in question or the enrichment of species could also reflect the differences in gut communities and their metabolic and genetic content [271].

BAs can also serve as markers for PC diagnosis. In a study, Xiong et al. identified eight metabolites in serum, including TCA, which could differentiate PC from the healthy controls and benign disease [272]. In a recent clinical trial study (NCT02531607), Navaneethan et al. demonstrated that volatile organic compounds present in bile could aid in the accurate distinguishing of PC from chronic pancreatitis [273]. However, the changing dynamics of individual BAs must be kept in mind while investigating their biomarker potential. A study in rats showed dynamic changes in BA composition throughout the enterohepatic circulation, and individual BAs had different circulatory system homeostasis dynamics. Furthermore, diet and circadian rhythm could impact BA homeostasis [274]. The gender-based circulating BA pool differences should also be considered while considering the biomarker potential of circulatory BAs [41].

The microbiota can also be used to predict responses to PC therapy. For example, a study in PDAC patients showed that bacterial lipopolysaccharide in pancreatic tumours was a negative predictor for adjuvant gemcitabine therapy [275].

Microbiota and their metabolites are the hotbeds for research in PC pathology, drug pharmacokinetics, therapy efficacy, and survival outcomes. Overall, the microbiota/bile acid/PC dynamics are complicated. Several factors affect microbiota–host dynamics, including lifestyle, diet, and disease pathology. The current studies portray the biphasic involvement of the gut microbiota/BA axis in PC. Given the important role of this axis in regulating host dynamics, more uniform and consistent approaches are required to increase the translation power of its applicability in a clinical setting.

## Figures and Tables

**Table 1:** Bile acid receptor expression levels and their association with pancreatic cancer development.

Bile Acid Receptors	Expression Level Findings	Key Study Findings for Association with Pancreatic Cancer Progression	References
FXR	Increased in PC tissues with lymph node metastasis	FXR overexpression in PC tissues with lymph node metastasis correlated with poor survival. Downregulation decreased proliferation and migration in PC cell lines	[108]
	Increased FXR and decreased histidine-rich glycoprotein (HRG) expression in PDAC tumours	Negative HRG and positive FXR correlated with TMM stages, invasion, metastasis, and poor prognosis in PDAC Overall survival time for FXR-positive patients or HRG-negative ones was significantly lower than negative FXR or positive HRG	[109]
		Patients with elevated FXR expression were associated with longer survival times compared to lower expression. Borderline association of high FXR expression and low histopathological grade	[110]
TGR	Increased expression in pancreatic cancer tissues compared to normal adjacent tissues	Elevated receptor expression correlated with an increase in tumour grade and lymph node metastasis	[111]
		TGR5-deficient mice demonstrated protection against pancreatitis upon exposure to bile acid	[112]
PXR	Elevated expression in PXR expression in PDAC patients presenting with increased tumour differentiation. Increased non-significant incidence of higher PXR expression in PDAC patients without lymph node metastasis	PXR expression did not correlate to survival. However, simultaneous overexpression of PXR with its co-receptors was associated with a less aggressive PDAC phenotype.	[113]
VDR	Increased expression (3-fold) in pancreatic cancer cell lines		[114]
	Increased receptor expression in endocrine islets in chronic pancreatitis and PDAC patients In PDAC patients, compared to the stroma, significantly higher expression in ductal and acinar cells	During PDAC development, the islets lose *CYP24A1* (gene targeted by VDR bound with vitamin D) expression, while the malignant cells increase expression.	[115]
	1.5-fold elevation of vitamin D in serum of PC patients (Egypt cohort)	The lower level of VDR-SNP or vitamin D is not a PC risk factor of the Egyptian cohort.	[116]
	Receptor expression in pancreatic cancer stroma	Serves as a transcriptional regulator of pancreatic stellate cells. Activation of stromal VDR overcomes chemoresistance. Gemcitabine in combination with VDR ligand improved survival in PC mouse models.	[117]
		Activation of VDR signalling can suppress the release of oncogenic miRNA from CAF-derived exosomes to inhibit pro-tumorigenic functions in PC cells.	[118]
		Association between improved overall survival outcomes and high VDR expression in PC patients.	[119]
		VDR signalling activation can reduce stemness in PC cancer cells.	[120]
	Abundant expression in highly differentiated tumour tissue compared to low or moderate differentiation	Low VDR expression correlated with poor PC prognosis.	[121]
		VDR variant rs2853564 was associated with overall survival in PC patients.	[122]
LXR	Abundant expression of LXRβ in PDAC patients		[123]
	Enriched LXR/RXR activation in the PC serum patients		[124]
	LXRβ expression and possibly abnormal localisation observed in PDAC patient tissues	The study showed LXRβ knockdown significantly decreased pancreatic cancer cell proliferation.	[125]
S1PR2		Taurocholic acid contributes to gemcitabine resistance via S1PR2 in pancreatic cancer.	[104]
